# What Is the Relationship between the Presence of Volatile Organic Compounds in Food and Drink Products and Multisensory Flavour Perception?

**DOI:** 10.3390/foods10071570

**Published:** 2021-07-06

**Authors:** Charles Spence

**Affiliations:** Department of Experimental Psychology, Anna Watts Building, University of Oxford, Oxford OX2 6BW, UK; charles.spence@psy.ox.ac.uk

**Keywords:** volatile organic compounds, aroma, flavour perception, multisensory, congruency

## Abstract

This narrative review examines the complex relationship that exists between the presence of specific configurations of volatile organic compounds (VOCs) in food and drink products and multisensory flavour perception. Advances in gas chromatography technology and mass spectrometry data analysis mean that it is easier than ever before to identify the unique chemical profile of a particular food or beverage item. Importantly, however, there is simply no one-to-one mapping between the presence of specific VOCs and the flavours that are perceived by the consumer. While the profile of VOCs in a particular product undoubtedly does tightly constrain the space of possible flavour experiences that a taster is likely to have, the gustatory and trigeminal components (i.e., sapid elements) in foods and beverages can also play a significant role in determining the actual flavour experience. Genetic differences add further variation to the range of multisensory flavour experiences that may be elicited by a given configuration of VOCs, while an individual’s prior tasting history has been shown to determine congruency relations (between olfaction and gustation) that, in turn, modulate the degree of oral referral, and ultimately flavour pleasantness, in the case of familiar foods and beverages.

## 1. Introduction

Multisensory flavour perception in undoubtedly a complex phenomenon including, as it does, the integration of gustatory, retronasal olfactory (see [1,2] on the distinction between the orthonasal and retronasal sense of smell), and, when present, also trigeminal cues [3,4]. In order to simplify matters, those working in the flavour and fragrance industries typically refer to the food aromas that they produce as flavours, based on the fact that the majority of taste is delivered by the volatile olfactory cues. Indeed, one can find the view that 75–95% of what people think that they are tasting actually comes from the sense of smell (i.e., from retronasal olfactory cues) echoed throughout the academic and popular press over the last three decades or so [5]. Though, as for so many often-repeated statistics, it turns out that there is no robust underpinning evidence concerning what the correct percentage might actually be in this case. Indeed, the relative contribution of different senses likely changes as a function of the specific food or beverage. Consider here only how the olfactory contribution to the multisensory tasting experience would appear to be much more pronounced in the case of a ripe Epoissé cheese than in the case of celery or sushi, say. As such, it is natural to think that chemical analysis techniques, such as gas chromatography and mass spectral data, that provide an analysis of the volatile organic compounds (VOCs) in food and beverage products, will herald a golden age for the understanding of sensory science. As Karakaya, Ulucan, and Turkan ([6], p. 179) put it: “*In the last two decades, improvements in materials, sensors and machine learning technologies have led to a rapid extension of electronic nose (EN) related research topics with diverse applications*.” Some commentators have even gone so far as to suggest that the “molecular composition of food dictates the sensation of flavour” ([7], p. 3; though note that this particular claim, which appeared in the preprint, did not make it into the final published article; see [8]). Here, I wish to take issue with this claim and with the more general suggestion that the VOC profile of a given food or beverage product allows one to predict precisely multisensory flavour perception (MFP). Specifically, this narrative review examines the complex relationship that exists between the presence of specific configurations of VOCs in food and drink products and MFP.

MFP involves the contribution of multiple sensory inputs, both volatile and non-volatile (i.e., sapid). What is more, gustatory and trigeminal inputs can sometimes play a surprisingly important role in determining the nature of the ultimate multisensory tasting experience that the consumer enjoys—both what they perceive, as well as where they happen to localize the experience. I first review the evidence concerning the suggestion that a relatively small number of key food odorants (KFOs) may play an especially important role in MFP in many of the foods we consume [9] (Section 2). Thereafter, I highlight some of the important ways in which the experience of taste (or rather flavour) is influenced by more than just a food or beverage product’s VOC profile (e.g., as measured by techniques such as gas chromatography, or the use of an e-nose; see Section 3). I will draw particular attention to the important role played by sapid (i.e., non-volatile) gustatory and trigeminal contributions to MFP [10]. I also summarize the evidence highlighting the important role played by individual differences, both genetic as well as differences in a taster’s prior exposure to particular flavours, in determining the nature of their flavour experience. Crucially, these various factors all contribute to the complex relationship that exists between the presence of specific configurations of VOCs in food and drink products and MFP.

In what follows, I will contrast the consumer’s perception of familiar and unfamiliar flavours/foods. This is an important distinction because while the predictive account of sensation based on VOCs can sometimes help to predict/describe aspects of the consumer’s perceptual response (such as perceived pleasantness or complexity) to novel/unfamiliar olfactory stimuli [11,12], more often than not it fails in the context of familiar odours/flavours. Our prior experience of flavours (specifically, combinations of tastants and odorants) has been shown to change our perception of the qualities of the latter. This is obviously highly relevant given that the flavours in the majority of food and drink products are likely to be more or less familiar to those who consume them.

### 1.1. Flavour Chemistry Dissociates from Flavour Perception

**Figure 1:**
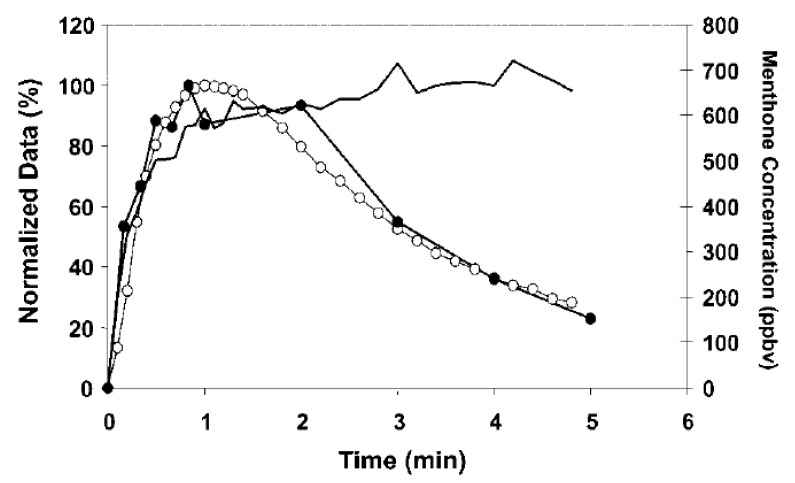
Sucrose release (filled black circles), menthone release (black line without symbols), and perceived intensity of overall mint flavor (Time-Intensity curve) (open circles), from a tablet type commercial chewing gum. The sucrose release data are the mean values from three panelists, while the menthone release and perceived intensity values are the mean of 11 panelists. Sucrose and perceived intensity values have been normalized for easy comparison. ppbv—parts per billion in the gas phase [Figure reprinted from Davidson et al. [13]].

A couple of examples from the field of flavour chemistry should be sufficient to highlight the difficulty associated with trying to predict MFP on the basis of the VOC profile of familiar product. In an intriguing study by Davidson et al. [13], the perceived intensity of menthol flavour (menthone) in commercial tablet and stick chewing gums was shown to be contingent on (that is, modulated by) the presence of sweet taste (sucrose) in the oral cavity (see Figure 1). The physical in-nose concentration of menthone (the relevant VOC), monitored on a breath-by-breath basis using direct gas phase atmospheric pressure chemical ionization-mass spectrometry, remained high over a period of several minutes. Swabs of saliva were taken from the tongue and analyzed using a rapid, direct liquid-mass spectrometry procedure. Surprisingly, the perceived intensity of the minty aroma actually tracked the intensity of sweetness on the palate, thus suggesting an important role for the sapid (or gustatory) element of MFP. Hence, while the presence of menthol (a VOC) was key to the nature/identity of the flavour experience that the participants in this study had, it was gustation (non-volatile sucrose) that was key to the participant’s awareness of the minty odour [14].

Meanwhile, Hort and Hollowood [15] reported on a study in which a sweet taste stimulus and fruity (banana) aroma were delivered at the same time to participants by means of a computer-controlled gustometer and olfactometer. When the two chemosensory stimuli were presented together, the participants reported an intense banana flavour (isoamyl acetate), just as one might have expected. Interestingly, however, the percept did not change substantially when the banana aroma was slowly phased out (i.e., leaving only the sucrose). By contrast, gradually eliminating the sweet taste resulted in the loss of banana sensation, despite the fact that the key VOCs associated with the banana aroma were still being presented. What such results, and others like them highlight, therefore, is the fact that while the profile of VOCs in a food or beverage product undoubtedly provides a meaningful constraint on what might be perceived (e.g., by the consumer), one often also needs to know something about the sapid contributions in order to gain a more complete understanding of MFP. Note here also that both mint and banana, the aromas used in the last two examples are both very familiar flavours in food and beverage products.

### 1.2. Is It Possible to Predict the Flavour/Aroma from the Molecular Structure?

There has been a great deal of interest in recent years around the question of whether knowing the chemical structure of a particular VOC can be used to predict odour perception [16]. For instance, according to one line of empirical research, hedonic judgments of specific unfamiliar VOCs have been shown to correlate with their molecular size, with larger molecules typically rated as smelling more pleasant [12,17]. What is more, as the size of unfamiliar monomolecular odorants increases (and thus the molecules become more structurally complex; [18]), the evidence suggests that people also generate a larger number of odour descriptors (this providing one measure of perceived complexity; [11,19]). While such results have been taken by some to suggest a meaningful link between molecular structure and multisensory odour perception, as Charles Sell noted some years ago, it is simply not possible to predict the odour qualities from the structure of molecules (that is, VOCs; [20]). Of course, one might wonder whether recent developments would lead Sell to change his tune [16]. There is also some relevant evidence suggesting that the pleasantness of combinations of odorants can be predicted on the basis of the pleasantness of the component odours [21].

The majority of the research has attempted to predict odour perception rather than flavour perception with the latter being rather more complex, and multisensory, than the former. It has also mostly involved unfamiliar isolated VOCs. As we will see later, predicting the nature and pleasantness of perceptual experience becomes much more difficult in the case of familiar food and beverage items typically comprising several hundred distinct VOCs. For instance, according to Maarse [22], apple juice contains something like 137 VOCs, while it has been estimated that complex food and beverage products such as fine wine or speciality coffee may contain as many as 600–1200 VOCs [23,24], p. 424, [25,26]). Also relevant to mention here, Dunkel et al. [9] note how food processing (e.g., fermentation, roasting) often delivers more chemically complex odour mixtures.

## 2. Key Flavour Odorants

It turns out, however, that only a subset of the VOCs present in the majority of food and beverage products are actually relevant to MFP. It is the subset of those volatile molecules that are detectable by the (average) human nose (and/or that exert an influence over what is detected) that matters for those interested in trying to predict MFP. According to Benzi [27], the latter constraint typically drops the relevant figures down to 30–40 VOCs detectable by the human nose that contribute in some meaningful way to flavour perception. Going one stage further, it has been suggested that there may, in fact, only be something like 226 KFOs across all foods, 16 of which have been found in 25% of all food and beverage products (see [9] for a list of KFOs). It is also worth bearing in mind that there may be a few chemosensory stimuli that can be discriminated without necessarily being perceived (such as has been suggested for fatty acid taste; [28,29,30]). As such, one might wonder whether there might not also be certain VOCs that do not have any perceptible qualities, and yet might be discriminable if absent.

However, even if the claim of a restricted range of KFOs is correct, that still does not necessarily tell us how a given food or beverage product will be perceived. This is because such chemical analysis of food does not say anything about the temperature at which a food is served which, as anyone who has tasted a warm cola drink or ice-cream will know only too well, can dramatically affect the taste experience [31]. When the latter food and drink items are served frozen/chilled, the sweetness will likely be perceived as just about right. However, these items taste unpalatably sweet when served warm. Note here that the chemical analysis of food or beverage products typically makes no reference to serving temperature [32].

### Complexity in Multisensory Flavour Perception 

Complexity is seen as a desirable quality in many food and beverage products (such as, for example, wine; [33]). As Singleton and Ough put it almost 60 years ago: “*Complexity has long been considered a desirable factor in the quality of most flavorsome or odorous products*” ([34], p. 189). As such, there has been considerable interest in the question of whether perceived complexity can be predicted on the basis of the chemical signature (or VOC profile) of a food or beverage product [35]. Might it be that the number of KFOs in a food or beverage product can be used to predict a consumer’s judgments of its complexity? Here though, once again, there turns out to be no one-to-one mapping between the number of VOCs, or even KFOs, and the complexity of MFP. Indeed, recent empirical research with a selection of specially chosen wines highlighted the fact that judgments of complexity are as much cognitive as perceptual [36]. So, while a flavour that delivers more perceptual elements is presumably more likely to be described as complex, sometimes a single unitary flavour experience may also be judged as complex, assuming that the taster knows something about the difficulty of generating that particular flavour. In other words, the complexity of a food or beverage product’s flavour is as much inferred as it is directly perceived. It is perhaps also worth noting that complexity is something that sometimes develops over the course of a tasting experience—again chemical analysis of VOC profile doesn’t necessarily provide any information about whether all perceptible elements will be available in the moment or will be experienced sequentially [35].

Some researchers have also questioned whether perceived complexity is necessarily the most relevant, or interesting, perceptual dimension. There has, for instance, been some interest in the alternative notion of ‘sensory intricacy’ [37]. The latter measure, which is distinct from complexity, extends across both olfactory and visual stimuli. In particular, more intricate sensory stimuli appear to generate more variable responses from people when asked to rate the stimuli using e.g., a range of semantic differential scales. 

## 3. Individual Differences in Multisensory Flavour Perception

Beyond the chemical composition of a food or beverage product, there are also several factors concerning the consumer/perceiver that can affect whether or not a given VOC is detected, and how it (or a particular combination of VOCs) is perceived (e.g., in terms of both its sensory-discriminative and hedonic qualities).

### 3.1. Genetic Differences in Multisensory Flavour Perception

It has long been recognized that we do not all live in the same taste world [38,39]. The presence of significant genetic differences means that the same VOC may be perceived in a qualitatively different manner or not at all (in the case of selective anosmias; [40]) by different consumers [41]. Consider here, for example, coriander leaf. The presence of the relevant VOCs in *Coriandrum sativum* mean that a taster will either perceive a pleasant, citrusy, herby taste, or else an unpleasant soapy taste. However, which of those perceptual responses (determined by the presence of the relevant VOCs) a given taster will experience depends on the taster’s genetic make-up [42,43]. Though, it is a little unclear from the literature whether coriander tastes soapy, smells soap, and/or has a soapy flavour. Androstenone, or boar taint, is another VOC that has been reported to elicit different perceptual responses amongst consumers [44,45]. One in every two people are unable to smell androstenone, an odorous steroid derived from testosterone; that is, they are anosmic to this particular volatile organic molecule. Meanwhile, 35% of the population find that it has a very powerful—and deeply unpleasant—stale, sweaty, urine smell (this is the reason why male pigs are castrated, i.e., to minimize the unpleasant aroma known as ‘boar taint’). Worse still, the individuals in this group tend to be exquisitely sensitive to this compound, with some being able to detect it at concentrations of less than 200 parts per trillion. The remaining 15% or so of the population report that androstenone smells sweetly floral, musky, and/or woody. At the same time, some consumers experience the smell as chemical-like [45,46,47].

As Lunde et al. [46] note, the human OR7D4 genotype predicts the sensory perception of meat containing androstenone and genetic variation in an odorant receptor significantly affects MFP and thus food preferences. Once again, one and the same VOC can deliver a handful of completely different perceptual experiences, depending on the genetic make-up of the taster. While androstenone and coriander are rare in generating such a wide range of qualitatively different perceptual responses, it is worth noting that we may all be anosmic to some number of olfactory stimuli, and individual differences in perceptual threshold for specific olfactants are relatively common. According to recent research by Trimmer and colleagues [48], genetic differences in olfactory receptor genes result in differences in ratings of pleasantness and intensity for a wide range of olfactory stimuli [49,50]. See Trimmer et al. [48] for a summary of the differing responses of consumers to various olfactory stimuli.

Coriander and androstenone are, then, just the tip of the iceberg as far as genetically determined differences are concerned. That is to say, every one of us is anosmic to some number of compounds, many of which are associated with food. So, for instance, our sensitivity to isovaleric acid (a distinctive sweaty note in cheese), s-ionone (a pleasant floral note added to many food and drink products; think of the fragrance of violets), isobutyraldehyde (which smells of malt) and *ci*s-3-hexen-1-ol (which gives food and drink a grassy note) all display significant genetic variation, while roughly 1% of the population are unable to smell vanilla [51,52,53,54,55,56]. What this means, in practice, is that there are some pretty profound individual differences in people’s ability to perceive these compounds.

A number of these genetically-determined selective anosmias in the population are undoubtedly relevant as far as the perception of food and drink are concerned [57,58,59]. Given the large number of selective anosmias, not to mention genetic differences in tasting, such as represented by an individual’s taster status [60], it is difficult to predict quite how a given consumer will perceive a given configuration of VOCs without also knowing something about their genetic make-up. One slightly more sophisticated question to ask here might be to ask how many of the KFO are subject to substantive genetic variation within the population. One might imagine, a priori, that such genetic differences would be less likely to occur for the more common KFOs (than for the less common ones), given that the perceptual response might be expected to influence the popularity of flavours. However, it is not obviously the case that that is true. Just consider the increasing popularity of cilantro/coriander leaf [61,62].

### 3.2. Congruency, Similarity, and Crossmodal Correspondences

Another factor that modulates various aspects of MFP relates to the congruency between the gustatory and olfactory components of flavour. In particular, congruent pairs of olfactory and gustatory stimuli have been shown to lead to increased oral referral of odorants to the mouth relative to when incongruent pairs of stimuli are presented [63,64,65,66]; see [67], for a review). So, for example, in one study, the oral referral of vanilla odour increased from 22% to 49% when a sucrose solution was presented to the tongue, while the oral referral of soy sauce aroma increased from 19% to 50% as a result of the presence of sodium chloride on the tongue [64]. There is a separate interesting question about the extent to which odorants also take on the relevant properties of those trigeminal stimuli with which they are paired. As Fondberg and colleagues [68] note, there is a significant relationship between congruency, pleasantness, and odour referral to the mouth [69]. Fondberg et al. created nine odour-taste stimulus pairs varying from maximally congruent (chicken odour with salty taste or citrus odour and sweet taste) to maximally incongruent (chicken odour and sweet taste or citrus odour and salty taste). The participants rated the pleasantness and localization of ensuing flavour sensation. Talking of the importance of olfactory-gustatory congruency to MFP obviously begs the question, though, of what determines congruency in the first place.

According to the literature, congruency is assumed to be acquired through experience. For instance, according to Lim and Johnson congruency refers to “*a taste that commonly appears with an odor in foods*” ([65], p. 288), mostly (if not entirely) results from associative learning “*the extent to which the two sensations are commonly experienced together in a food*”, following prior exposure to the component stimuli when presented together in flavour stimuli. One important point to note here is that pairs of gustatory and olfactory stimuli that commonly co-occur in food and beverage products not only become more congruent over time, they may also become more perceptually similar. That is, specific food aromas come to take on certain of the taste properties with which they are frequently/commonly associated [70].

According to a substantial body of research from Stevenson and his colleagues, within just a handful of co-exposures to a novel food odorant and a given tastant, even if presented at a sub-threshold level and/or unattended [71], the odorant with start to take on the taste properties of the latter [72,73,74]. That said, recent research from Fondberg et al. [75] surprisingly failed to demonstrate any acquisition of taste properties by olfactory stimuli (basil and orange flower). These researchers conducted a study in which one of the odours was exposed with sucrose for five days in a chewing gum whereas other was exposed without sucrose. However, no change in rated sweetness of the odorant as a result of associative conditioning was observed. That said, chewing gum is a particularly unusual foodstuff inasmuch as it does not change volume/structure no matter how long you chew it, and if not removed from the mouth at the appropriate time, it may end up delivering oral-somatosensory stimulation in the absence of gustatory and/or retronasal olfaction.

Here, though, it should be stressed that not everyone even believes in the possibility of making similarity judgments across the senses. For instance, the eminent early German psychophysicist, Hermann Helmholtz, argued long ago that: “*The distinctions among sensations which belong to different modalities, such as the differences among blue, warm, sweet, and high-pitched, are so fundamental as to exclude any possible transition from one modality to another and any relationship of greater or less similarity. For example, one cannot ask whether sweet is more like red or more like blue. Comparisons are possible only within each modality; we can cross over from blue through violet and carmine to scarlet, for example, and we can say that yellow is more like orange than like blue!*” ([76], p. 77). 

Interestingly, however, Helmholtz does not mention olfaction, or flavour perception. Whether or not Helmholtz’s general claim is correct, it can be argued that one can make meaningful similarity judgments between olfactory and gustatory stimuli. Certainly, participants would appear to be able to assess the degree of similarity of various combinations of olfactory and gustatory stimuli [77,78], in part due to the acquired taste properties of olfactory stimuli [70,77]. 

Consider here only how most of us perceive vanilla to smell sweet, despite the fact that vanilla pods are actually very bitter to taste [79]. The acquisition of taste properties by odorants means that food aromas can, over time, sometimes end-up becoming more similar to the tastes with which they are commonly associated, or paired [77,78]. As a result, people are able to make meaningful judgments of the similarity of pure tastants and olfactory stimuli (e.g., as when judging the similarity of sugar to various aromatic spices in an intriguing study by Blank and Mattes [77]), thus seemingly contradicting Helmholtz’s general claim. It is worth stressing that this phenomenon is unique to pairing of olfactory and gustatory stimuli (e.g., while people often learn that certain colours predict specific flavours, they do not become more similar over time; see [80]). At the same time, this can lead to cultural differences in flavour perception given the different combinations of ingredients/flavours that are such a distinctive feature of the cuisines of different cultures [81,82].

It is important at this point to try and distinguish between the seemingly similar notions of perceptual similarity and congruency. The phenomenon of acquired taste properties leads both to an increase in congruency and to an increase in perceived similarity. But note that similarity does not necessarily lead to a pleasing combination, nor does it lead to increased oral referral [83]. Acetic acid and citric acid are similar (in that they are both sour tasting). However, they are not necessarily congruent, in that they do not necessarily co-occur in food and drink [84]. Meanwhile, combinations of basic tastes such as bittersweet or sweet-and-sour co-occur without necessarily being judged as increasingly similar over time. Indeed, these taste pairs are often put in opposition, rightly or wrongly, on taste scales [85,86], so pairings of tastants do not become more similar, only combinations of taste and smell. Similarly, colours correspond crossmodally with tastes and flavours as a result of co-exposure without necessarily becoming more similar [80].

The key point to note, though, is that MFP is influenced by familiarity [87,88], especially for those VOCs that have regularly been paired with tastants previously [89]. Attention may also be directed somewhat differently when a consumer experiences congruent and incongruent combinations of taste and smell [83], in part due to the former congruent combinations being more likely to be perceived as a perceptual gestalt [67,68,90,91]. The acquisition of taste properties by odours is part of the reason as to why it can be especially hard to predict how consumers perceive in the case of food and drink products. Returning to the fundamental question addressed by this narrative review, the variable experience of congruency and/or similarity between gustatory and olfactory cues is part of what makes it so hard to predict MFP exactly based on nothing more that the VOC profile of a given food or beverage product.

### 3.3. Expertise

Flavour experts, be it chefs [92], or wine experts [93], may also be differentially able to distinguish olfactory versus gustatory contributions to flavour when tasting food and beverage products, or at least those that happen to fall within their area of expertise. To the extent that such a claim is correct, this will also mean another potentially important individual difference in how the VOCs in food and beverage products are perceived [15]. Such differences may once again reflect differences in the allocation of attention amongst the chemosensory modalities during tasting [71,90].

## 4. On the Use of Electronic Noses and Tongues

Ultimately, given the problems with predicting MFP based solely on the VOC profile of a given food or beverage product as outlined here, it is your author’s opinion that the latest generation of electronic noses and tongues ([94]; see [6] for a recent review), some of which can now fit on a chip; [95], will likely play a more important role in the detection of faults, such as the presence of cork taint in wine [96,97], than necessarily in predicting MFP. Though, even here, figuring out what an acceptable threshold is for cork taint will require careful sensory testing amongst the target population given that threshold to detect this compound, 2,4,6-trichloroanisole; [98] differs widely between individuals [99] and also varies as a function of wine style ([100]; Your author is fortunately anosmic to this compound). Researchers from Intel working together with olfactory neurophysiologists from Cornell University successfully built a system using Intel’s Loihi neuromorphic chip to process the data from an array of chemical sensors. This “electronic nose” system can detect up to ten different chemicals as accurately as a state-of-the art deep learning system, but with very little training required.

## 5. Conclusions

The analysis of VOCs in food and drink has undoubtedly made great progress in recent years. However, attempting to use such chemical signatures (or VOC profile) to infer perceptual qualities has, by-and-large, failed. While chemical description can be used to infer perceptual consequences in a small number of experimental conditions, typically with constrained olfactory stimuli that are unfamiliar to the participants, that is rarely the right description for the majority of the flavourful stimuli that we are familiar with. The problem when it comes to trying to predict MFP is that genetics, prior experience, expertise etc. all mean that it can be hard to know how an individual will experience a given VOC profile. Although the perceptual quality when tasting is largely determined by the presence of VOCs, the importance of gustatory cues, and even of the congruency between olfactory and gustatory cues has been shown to exert a profound influence over the final tasting experience, including both where the experience is localized as originating (i.e., the phenomenon of oral referral), and how pleasant it is rated as being—though these appear to be two distinct processes. Cultural influences on the congruency and similarity of different tastes and flavours, as well as the differing patterns of attentional focus (e.g., in flavour experts), also mean that the relationship between MFP and the VOC profile of a given food or drink product is likely more complex (and somewhat unpredictable) than is sometimes realized by flavour chemists and computational gastronomists.
